# The Personal and Professional Impact of Patients’ Complaints on Doctors—A Qualitative Approach

**DOI:** 10.3390/ijerph19010562

**Published:** 2022-01-05

**Authors:** Bianca Hanganu, Beatrice Gabriela Ioan

**Affiliations:** Legal Medicine Department, Faculty of Medicine, Grigore T. Popa University of Medicine and Pharmacy of Iasi, 700115 Iasi, Romania; beatrice.ioan@umfiasi.ro

**Keywords:** complaints, personal impact, professional impact, qualitative research, medical practice, doctors, patients, semi-structured interview

## Abstract

*Background*: Complaints regarding medical practice represent a harsh reality of the current world. Patients have the right to receive explanations and compensation when they are injured during the medical act, but the increased potential for exposure to complaints determines personal and professional consequences for the doctors, with significant impact on their health and practice. Thus, the aim of our research was to analyze in depth the impact of complaints on the doctors involved. *Materials and methods*: The authors conducted a qualitative study, using a semi-structured interview, addressed to doctors who had complaints from patients. The participants in our research were identified using an adapted version of the snowball method. *Results*: After the analysis of the interviews using the inductive method, nine themes resulted, seven of which are addressed in this paper: injustice, personal impact, professional impact, difficulties, supportive factors, the attitude of the hospital management and the attitude of colleagues. At the personal level, the doctors were overwhelmed by insomnia, nightmares, stress and anxiety, and at the professional level by doubts about medical decisions, fear, anxiety and the tendency to avoid patients with severe diseases. *Conclusions*: The study revealed that physicians who had complaints from patients are deeply affected by the complaint itself and the associated investigation procedure, even if, to a lesser extent, some of the participants found motivation for a better management of the situation. The study also showed the need for changes in the legal and medical systems in order to create mechanisms to support the doctors during the investigation process.

## 1. Introduction

Success in medicine is the result of the harmonious combination of several elements: technological progress (with increasingly advanced possibilities for diagnosis and treatment), the allocation of appropriate resources to medical institutions, theoretical and practical training of physicians in accordance with this progress and patients’ characteristics—especially regarding their adherence to therapy. To these are added the doctor–patient relationship, especially the way one relates to the other, and the unity of the medical team, both in terms of the relationship between its members and their relationship with the patient [1].

On the one hand, it is important and fair for patients (or their families) to have the opportunity to make complaints [2,3,4,5] in an attempt to receive explanations and compensation when their lives or bodily or moral integrity are harmed by a medical act [6]. The purpose of the complaints and investigations launched consecutively is precisely to ensure the development of medical practice in optimal conditions [7], protecting the patients [2,6,8,9] and maintaining clinical standards at an appropriate level [4,6,9]. Many of the changes aimed at improving the healthcare system have as their starting point the level of patient satisfaction [3], taking into account their dissatisfaction with the experience with the medical institution and with the professionals in the field [10].

On the other hand, however, the significant potential for exposure to complaints determines inevitable personal and professional consequences for the doctors [3,11], which should not be neglected. Stress is a quasi-constant element in medical practice, by the nature of the profession, even if it does not feel equally strong in all specialties. Stressed doctors are at risk of reduced professional performance, with repercussions on patient care [12]. The worrying increase in the number of complaints from patients creates significant additional stress for practitioners [8], augmented sometimes by the fact that these conflicts are initiated by external factors, such as people—even healthcare professionals, who encourage the patient to complain. Although medical training involves—in addition to the assimilation of knowledge and the development of practical skills—endurance in difficult working conditions, under stress and often under the pressure of time, complaints from patients often surprise doctors unprepared and have traumatic potential [13].

The doctors’ well-being and the quality of care they provide to patients are interdependent [14,15]. Therefore, in order to be able to carry out the activity at the highest standards, the internal and the external condition of the doctor must be in balance. The direct consequences of complaints on the doctors concern on the one hand their inner space, through the psychological suffering [2,5,10] associated with the process of investigating the complaints, and on the other hand their outer space, related to practice, through which doctors connect with their patients [10]. Data from the literature show the negative influence that patients’ complaints may have on the well-being of doctors [2,10,16], with their health sometimes being harmed in the long term [16]. Analyzing the situation from this perspective, it can be seen that there is a risk that the complaints system will cause more harm than good to patient care [4] by directly affecting the doctor and the important components of the doctor–patient relationship [17].

Given the fact that the experience of complaints may have a significant negative impact on doctors’ health and neglecting it could have serious consequences—such as psychological disorders, loss of job satisfaction and changes in practice [10]—the aim of our research is to analyze in depth the impact of complaints on the doctors involved. Thus, we aimed to investigate how doctors experience complaints and how complaint management could be improved, in order to prevent their negative impact on doctors and to prevent them from occurring.

## 2. Materials and Methods

### 2.1. Instrument and Data Collection

In order to identify the impact of complaints on the doctors involved, by analyzing their experiences, attitudes, emotional state and feelings we conducted qualitative research. This type of research, which is based on discussions and/or observations, allows the researcher to penetrate the depth of a phenomenon and obtain as many details as possible [18], without aiming to generalize the results [18,19].

Data collection was performed using a semi-structured interview applied individually. The interview guide was prepared by consulting the relevant literature on the topic [5,13,16,17,20,21,22], followed by free discussions in brainstorming sessions between the authors of this paper. Based on the obtained data, we made a list of the most relevant issues that could be addressed in the interviews and then included them in the structure of the interview in the form of open-ended questions, through which participants had the opportunity to relate freely to the experiences they had during the investigation of the complaint and after the settlement of the case. The interview guide included 28 questions, structured in 4 sections: (1) 2 questions for accommodation, through which we invited participants to reflect on the reality of malpractice complaints and the current level of risk of complaints from patients in Romania; (2) 15 questions strictly related to the impact of the complaint on the doctor immediately after receiving the notification and during the investigation of the complaint; (3) 7 questions to find out how the complaint and the associated process of investigation subsequently were reflected in the personal and professional life of the doctors; (4) 4 questions that allowed discussions about possible solutions or elements that could have been helpful to doctors during the investigation.

The interviews were conducted in May 2020 by the first author of this article, either face-to-face or by phone, through *WhatsApp*, which allowed a video call (to overcome challenges imposed by the COVID-19 pandemic). This interview was the first interaction of the author with the doctors participating in the study. At the beginning of the interviews, we requested consent for recording, we provided information about the research project, and each doctor signed an informed consent form to participate in the study. Likewise, participants had the opportunity to withdraw from the study and cancel the interview at any time. The structure of the interview was the same for all participants, but depending on their answers, the questions had one course or another, sometimes with additional questions to clarify certain issues.

### 2.2. Recruitment of Participants

The target population was represented by doctors from different specialties, of different ages, both men and women, who personally experienced a complaint from patients regarding any of the aspects of professional liability: civil, disciplinary or criminal.

Unlike some countries (e.g., The Netherlands) [5], in Romania there is no public database in which to record details identifying doctors who have been involved in complaints from patients, which would have facilitated researchers’ access to the target population. Therefore, in order to be able to identify the participants in the study, we adapted the snowball method. This selection method is useful for situations in which the target population is part of a difficult-to-access category and involves identifying and recruiting participants through acquaintances. The number of study participants is not exact, recruitment taking place in a step-by-step manner until data saturation is achieved, i.e., when the conclusion is reached that adding new participants would not bring new information relevant to the study [18].

### 2.3. Analysis

All the interviews were transcribed literally, after listening to the recordings. For the qualitative analysis of the content obtained from the interviews, the authors used the inductive method: all the transcripts were read individually by each author repeatedly in order to identify the common elements that would help to generate the themes. Then, the authors transformed into codes the words frequently used by doctors to describe their experiences. After individually making a coding scheme, the authors analyzed the identified codes together, and where they identified discrepancies, these were discussed so that in the end a consensus was reached. The codes thus identified were used to establish themes and subthemes.

N.B.: The interview was semi-structured, following a guide with pre-set questions, but given that the discussions took place freely, with interventions and returns, we performed a qualitative analysis of them (codes, themes and subthemes) as a whole.

### 2.4. Ethical Approval

This study was approved by the Research Ethics Commission of the Grigore T. Popa University of Medicine and Pharmacy of Iasi, no. 16434/30.07.2019.

## 3. Results

A total of nine doctors from different medical, surgical and paraclinical specialties were interviewed. The study group included four men and five women who work in different cities in Romania. The complaints faced by the interviewed doctors concerned all three aspects of professional liability (i.e., civil, criminal and disciplinary), isolated or combined.

The interview lasted an average of 38 min, with extremes of 20 min and 75 min. In order to ensure the confidentiality of the data, we coded all the participants, assigning to each one a number in the order in which the interviews were conducted, preceded by the letter P.

We identified nine themes, seven of which are the subject of this article: injustice, personal impact, professional impact, difficulties, supportive factors, the attitude of hospital management and the attitude of colleagues. The other two themes were: assessing the risk of malpractice complaints in Romania (addressed in a previous article [23]) and suggestions for preventing complaints and limiting their impact on the physician who received a complaint.

### 3.1. Injustice

In general, the participants experienced a feeling of injustice because of the complaints, motivated by issues related to patients, as well as issues related to medical and juridical practice.

#### 3.1.1. Issues Related to Patients

Doctors have often mentioned financial motivations behind complaints, i.e., patients’ desire to obtain money effortlessly:

*And here I felt from the very beginning the desire of the claimant to earn some money.*

(P6)

*His son […] he wanted to get rich at my expense.*

(P9)

Another reason frequently mentioned was the patients’ low level of medical education—which does not allow them to appropriately understand medical terms, procedures or prioritization of the medical services. Moreover, the patients or their families often complained before asking the doctors for explanations:

*The patient’s daughter complained against me and the resident doctor that—finally, as is usually the case, that I didn’t do anything to her father, that I didn’t do what she thought should be done, including fibrobronchoscopy—although there was no indication, and that I did not send him to cardiology—because she saw the cardio-respiratory arrest written on his death certificate and considered that I have failed to diagnose a heart condition.*

(P1)

*Patients or relatives, dissatisfied […] of the fact that they have long awaited in the emergency unit, they do not understand that emergency cases have priority. Consultations more or less of family medicine have little to look for in the emergency unit.*

(P5)

In addition, due to the patients’ low level of medical education, some doctors discussed the fact that patients do not always follow the medical recommendation or they postpone the presentation to the doctor:

*[Patient] did not come for check-up for two years.*

(P9)

#### 3.1.2. Issues Related to the Medical Practice

Some doctors experienced a feeling of injustice because they received complaints despite the fact that they had been used all their knowledge and abilities to solve the respective cases, following all the existing protocols and recommendations:

*According to all the procedures, treatment and diagnostic guidelines, I did everything—and I usually did everything for these patients and I didn’t do anything wrong, and yet I was complained about.*

(P1)

The feeling of injustice was also related to the complexity of the case and the difficulty of treating the patient’s disease:

*Because, unfortunately, for all the surgical interventions with the complexity of the one I performed and for the clinical and intraoperative situation that the respective patient presented, there was the possibility of such complications. Because, virtually, all major surgeries have a percentage of intraoperative and postoperative complications that are cited in the literature.*

(P8)

The superficiality of the colleagues, doctors involved in resolving or managing the complaints, was also the motivation for the feeling of injustice for many of the interviewed doctors.

*[…] the superficiality of my colleagues from the Public Health Directorate and the Ethics Commission of the hospital who condemned me even though they did not ask me anything at all.*

(P4)

#### 3.1.3. Issues Related to the Juridical Practice

At the same time, doctors pointed out the shortcomings and superficiality of the juridical system, with specialists who have no knowledge of medical terminology:

*You realize that there are things that no one in the court has the ability and time and goodwill to analyze in detail and… They simply take them as such… You can keep saying that is not the pathologist who indicates the treatment, that it is a commission that was not established—oncology, multidisciplinary commission. Nobody cares. And that’s how you lose.*

(P6)

### 3.2. Personal Impact

#### 3.2.1. Negative Inner Feelings

The personal impact immediately after the complaint was dominated among most of the doctors by negative inner feelings of concern, anxiety and stress. Concern and anxiety were linked, for example, to the negative consequences of a potential unjust guilty verdict:

*I was only thinking about this thing. I was only thinking about the continuation of the injustice and somehow, if, I don’t know, that committee considers in any way that I made a mistake, I was only thinking about the totally unfair repercussions that could have appeared to me in conditions where I really didn’t do anything wrong. And these repercussions could be, well, quite important.*

(P1)

The factors that induced an increased level of stress in the doctors who received complaints were related either to the presentation to the commissions that analyzed the complaints, with the need to offer explanations, or to the presentation in court:

*And that stress of going to the commissions, and explanations, and…*

(P1)

*The names of all the people who have a trial appear on the door, on a screen. And next to my name—“tort”. It matters to me […]; to me that word represented a fantastic trauma. To see it every month… tort. No, it is not easy, I do not wish that to anyone.*

(P6)

#### 3.2.2. Relation with the Family

Some of the participants received important support from their families:

*It mattered a lot the support I received from my wife.*

(P1)

But two of the doctors mentioned the negative effect on their marriages, suffering the disappointment of separating from the life partner:

*A divorce also happened during that period…*

(P9)

#### 3.2.3. Rest Turmoil

During the investigations, all these negative inner feelings also resulted in rest turmoil, many of the doctors stating that they experienced nightmares or insomnia:

*I had some pretty shallow nights… my mind was always there.*

(P9)

#### 3.2.4. Perpetual Concern

Some of the doctors reported that despite the long time since the complaint, their personal lives continued to be dominated by the quasi-constant concern about current cases, with the impossibility of detachment and the interruption of rest during the night:

*[Personal life] It will never be the same. Because I will always bring home problems from the hospital that are amplified by that moment. Significantly more. I mean, I’ve never been able to relax at home or on vacation, without thinking about every patient I’ve seen. If I did everything. And I even have moments when I wake up at night and think: did I take tests on that patient…?!*

(P1)

### 3.3. Professional Impact

#### 3.3.1. Negative Inner Feelings

Negative inner feelings were also described in connection with the impact of complaints on the current professional activity that is dominated by feelings of distrust or insecurity regarding their own medical decisions:

*I ended up questioning every therapeutic decision.*

(P1)

Some participants reported feelings of fear or avoidance during their professional activity:

*After the trial, I became a fearful man. I’m afraid to intervene alone, to jump into major interventions.*

(P9)

*[…] I really didn’t want to perform on-call shifts anymore, you know?! I looked for all sorts of avoidance ways, so to speak.*

(P1)

#### 3.3.2. Defensive Medical Practice

For some of the doctors, defensive medical practice was a method of protection during and after the investigations, sometimes as a consequence of the feeling of insecurity on the medical decisions. Defensive medical practice was reflected in the following:

- Requests for additional unnecessary examinations

*This also affected the relationship with colleagues from other hospitals, because I had come to ask for a cardiology consultation where it was not even the case.*

(P1)

*You see a child that you have to operate today. A trivial surgical intervention. Who does not need a lung x-ray, does not need a pediatric consultation, because the child has nothing else. But you have to do these extra things. Just to cover yourself…*

(P4)

- Reducing the complexity of surgical interventions or reducing the work schedule and the number of patients

*In cases of severe complexity, I gave up the execution of extensive surgical interventions for the same type of pathology.*

(P8)

*I try to work less, I try not to perform too many operations so that I can follow things better. Much more… Much more carefully. Much fewer interventions. Fewer patients.*

(P9)

- Avoiding collaboration with resident doctors

*[Now] I enter with other colleagues… On that patient I operated with a resident doctor. A rather difficult intervention […] many complications can occur. Now I enter the operating room with a specialist or senior colleague.*

(P9)

- Thoughts of changing the profession:

*I rather get started and do something else.*

(P3)

#### 3.3.3. Positive Attitude

Although most of the participants reported the negative impact of the complaints, two of the interviewed doctors had a positive attitude, were confident in the decisions they made and did not report a major professional impact from the complaint.

*At that time it did not affect me in any way because I knew that I had done everything humanly possible in that case and I cannot say that it affected me professionally.*

(P5)

For another doctor, the previous complaints made it easy to approach with detachment such actions on the part of patients:

*I’ve had some previous allegations of malpractice. And of course I now look at these cases with a completely different experience and a much higher degree of detachment, because in all these allegations of malpractice I do not blame myself for anything, medically and surgically.*

(P8)

#### 3.3.4. Improving Communication with the Patient

A major change described by participants in medical practice after experiencing the complaint was related to the doctor–patient relationship, with improved communication, increased attention to the words used and clear calibration of expectations of patients and family members, as well as greater attention to the informative part of the medical act:

*First and foremost I changed the communication and the clear calibration of the expectations of the relatives.*

(P1)

*I’m more careful when patients sign [informed] consent. I’ve never been careful with that before.*

(P2)

#### 3.3.5. Additional Records

Most of the doctors stated that as a result of the complaint they became excessively cautious and recorded more information in the medical documents in order to be protected in the future:

*Many times now you are forced to be a better bureaucrat than a better professional. This is simply what malpractice teaches you.*

(P4)

### 3.4. Difficulties

During the investigations, the doctors faced a series of difficulties that increased the negative effect of the complaint. These difficulties were related to both the juridical and the medical systems.

#### 3.4.1. Difficulties Related to the Juridical System

The lack of knowledge of medical terms among the specialists from the juridical system was reported as a difficulty by two of the interviewed doctors. This difficulty was all the more important as the representation of their cases in court depended on this knowledge:

*And… and the specialty domain is unknown, and is so difficult also in the court to expose your problem. And the lawyer… for five years… forgets the terms from trial to trial … And what can I tell you…? The judges don’t even know how to say adenocarcinoma—they say ade…nomo…carcinoma and their tongues are tangled in their mouths.*

(P6)

#### 3.4.2. Difficulties Related to the Medical System

- Complaint investigation process

Some of the participants experienced negatively the hearing by the members of the disciplinary commission and of the commission within the Directorates of Public Health, referring to the way they were treated or to the fact that the members of the commission made a decision without giving the doctor a chance to be heard:

*Ad-hoc commissions had to be formed to judge you, you are not called to this trial, no one tells you anything, the next day you see written in the newspaper: Dr. X is guilty of killing someone!*

(P4)

Many participants reported that the process of investigating the complaint was generally a resource-consumer, in terms of money, time or energy:

*So they are very long, very winding, very cumbersome and expensive procedures.*

(P6)

b. The superficiality of experts

Another element of difficulty was the superficiality of the experts appointed to analyze the case:

*[…] When there are two different expertise, with different conclusions, then the higher institution should have explained to us with one sentence, two, three, why one is valid and the other is not valid. Which didn’t happen.*

(P6)

c. Lack of support

For some of the doctors, another difficulty was the lack of support from colleagues and from the management of the medical institution:

*If you have a problem, you have to solve it yourself. The hospital doesn’t help you, nor do your colleagues. You alone have to solve everything.*

(P9)

### 3.5. Supporting Factors

The doctors also talked about what helped them get over the investigation more easily. In this regard, the participants mentioned both elements related to medical practice and the support they received from family or lawyers.

#### 3.5.1. Factors Related to Medical Practice

- Continuation of professional activity and therapeutic success

Most doctors referred to the importance of continuing their professional activity and the therapeutic successes of that period for regaining self-confidence altered by the complaint:

*A very big advantage was the fact that I continued to practice and I think that the confidence in my professional attitude, or, well, in, in my professional skills was brought by cases that I treated in the hospital during that period.*

(P1)

b. Confidence in their own medical conduct

For some of the doctors, the belief that they had done the right thing in the decisions they made was a fulcrum, an element that helped them detach from the thought of the complaint:

*I knew that if the patient would have come to operate on her again, I would have done absolutely the same. Because I followed absolutely all the protocols and all the recommendations of the guides and protocols that exist in Romania or anywhere in the world about that type of medical pathology.*

(P8)

#### 3.5.2. Family

The support of family was also mentioned, either by the possibility of detachment from the preoccupation induced by the investigation with the extended family, or by the trust that the life partners offered:

*What helped me the most through was my family. My wife who believed in me.*

(P4)

#### 3.5.3. Lawyers

Some of the doctors appreciated the support of lawyers, who gave them confidence and fought alongside them:

*And in the end the lawyer somehow believed in me and we fought together and justice was finally served.*

(P4)

### 3.6. Attitude of Hospital Management (Neutral, Positive, Negative)

Regarding the attitude of hospital management, the doctors’ testimonies were divided. Most said that their hospital management had a neutral attitude, in the sense that those in charge simply responded to the requests of the investigative bodies by making available the copies of medical documents necessary for the investigation and by statements in which they noted that the protocols were followed:

*The hospital had a neutral attitude. I mean… you had that complaint and the manager and medical director… sent copies… and said that the hospital‘s procedures were followed.*

(P1)

Only two doctors said they benefited from the support of the hospital’s lawyer, but in both cases the complaint concerned the institution as well. Moreover, one of the doctors spoke about the appreciation of the management towards their professional activity:

*I had no repercussions. Moreover, I was kept in office, my activity was extended. So from this point of view I had no problem. The hospital and the management of the hospital have appreciated my work for thirty years.*

(P6)

Two of the participating doctors experienced difficulties on the part of the management. They talked about the restriction of their activity and about the difficulties they had to go through:

*[They didn’t offer to me] any help. I am sorry to say. On the contrary. They took the worst measures for me—restricting my activity.*

(P2)

### 3.7. Attitude of Colleagues (Neutral, Positive, Negative, Curiosity)

From the participants’ reports, it appears that the attitudes of colleagues during the investigation were varied and covered a wide range that included neutral, positive or negative attitudes or were dominated by curiosity.

*The attitude of the colleagues was of curiosity. To know what are the investigation steps within the College of Physicians or what are the investigation steps by carrying out this legal action.*

(P8)

In addition, given the increased risk of some medical specialties, one of the participants said that his experience induced fear in colleagues, all of whom ended up carrying out their medical activity in a defensive manner:

*[…] it was as if my fear clung to them. So we started practicing some kind of defensive medicine. So we seem to be afraid of the patients now.*

(P9)

## 4. Discussion

In this study, we aimed to explore the impact of the patients’ complaints on the physicians involved, both in terms of personal life and medical practice.

The well-being of the doctor is reflected in his/her professional activity, so that the personal impact cannot be completely separated from the professional impact. The doctor’s emotional status, his/her attitude towards work and patients and his/her ability to cope with professional stress, influence his/her ability to provide high-quality care. [17]. Complaints from patients or their families are a source of a massive avalanche of emotions for doctors around the world [20]. Thus, the subject of negative emotions is common to our study and studies published in the literature, doctors most often referring to experiencing stress. For example, Guest et al. [24] reported following their study on 72 oncology surgeons, 50 of whom had previous complaints, that the complaint was a major source of stress for more than half of those involved. Stress can influence both personal and professional life, risking loss of job satisfaction, as found in the study published by Jain and Ogden [16]. Elevated stress levels can also interfere with physicians’ professional performance by affecting mental processes such as memory, concentration and attention, by increasing irritability and decreasing decision-making capacity [12]. We understand, therefore, that it is fundamental for doctors to have a state of well-being that allows them to take care of patients’ safely [17].

Similar to our study, the theme of injustice was identified in the study performed by Verhoef et al. [5], who found the feeling of injustice in connection with the investigation procedures, with the way in which some of the doctors were treated by the members of the disciplinary commissions, the investigated doctors questioning the methods, expertise and judgment of the commission members or even feeling that they were treated from the beginning as guilty. In our study, we identified two doctors who felt deeply affected by the way they were treated by the members of the commissions of investigation, one who felt treated as guilty from the beginning and the other who was sanctioned by one of the commissions without even being heard. In addition, Bourne et al. [3] reported, based on the results of their study on 7926 doctors in the United Kingdom, that doctors investigated by the General College of Physicians were affected by the way they were approached by members of the commissions that analyzed the complaints, experiencing feelings of negligence and betrayal. Regarding the attitude of patients or family, various authors have highlighted the role of poor medical education in a patient’s interpretation of the negative outcome of the medical act [10] or lack of understanding of the limits of medicine [25], while other studies have referred to financial motivations behind complaints [26,27,28,29].

Regarding the injustice felt in relation to the legal procedure, Santoro [28] talks about a common struggle of the press and the legal system to hunt down those harming the patients while forgetting that they also have certain responsibilities. An adaptation of the proverb “See the straw in someone else’s eye, but do not see the beam in your own eye” means in this case that justice should support doctors by providing a legal framework that defines more clearly the terminology regarding the professional negligence, that provides solutions against the abuse of unfounded complaints, that shortens the duration of the trials and that takes into account also the liability of legal professionals [28].

The participants in our study highlighted the theme of family life from two perspectives. A first perspective was the impact of the complaint on the doctor’s family life. The personal life of the doctor involved in a complaint can be affected both by his/her inner feelings and by the repercussions of the negative emotions on family life [16]. When things do not go well in the family, professional activity will suffer, and at the same time, the stress at work will be brought home, with the risk to negatively influence the couple’s relationship [12]. The latter aspect is highlighted in literature [16] as well in our study, as two of the doctors stated that complaints affected family life, leading to divorce, which can be seen as an indirect consequence of a complaint. A second perspective was represented by the supportive role of the family in overcoming the feelings triggered by the complaint, and most of the doctors in our study found support inside their families.

In the context of loneliness, we can include the feeling of abandonment from colleagues and hospital management, a theme also common in our study and other studies published in the literature [16], in which participants felt the lack of support from their colleagues. On the other hand, both in our study and in other studies published in the literature there were participants who felt the support of colleagues and hospital management, the feeling of loneliness being isolated [5]. The involvement of hospital management in the process of resolving complaints can be very helpful in reducing the impact of complaints on doctors [5,21], both by providing juridical support (lawyers to represent the doctors in court or to guide them on the procedures), as well as by creating a blame-free working environment so that doctors do not fear disclosing the occurrence of an incident [21].

Other results of our study consistent with the results of studies published in the literature are those related to the appearance of the accused doctor in court, which is extremely stressful and potentially traumatic [14]. It is important to note that the doctor often pays this personal price regardless of how the investigation is resolved [20]. The stigma, stress and moral damage of being sued, of being effectively involved in the judicial procedure, including appearing before the court panel, remain embedded in the lives of the doctors even if they are acquitted [11,13,14,28].

Studies also show that sometimes the emotions of the accused doctors are contradictory, with a shift from doubt to anger, frustration and defiance, with shock and panic or feelings of indignation towards patients in general [16]. Moreover, these doctors risk living with depression, feelings of anger [16,17], guilt and shame both during the investigations and afterwards, for a long period [5,17]. We identified similar results in the study conducted by Reed et al. [30], in which 86% of the participating doctors stated that the negative effects of the complaint are felt for a long period of time in the career of the doctor involved. For the doctors participating in our study, the personal impact was major immediately after the complaint and during the investigation. There is a balance between the responses of those who managed to overcome the event and those who continue to be personally disturbed. Verhoef et al. [5] pointed out the possibility of the persistence of the impact of disciplinary complaints long after the end of the investigation process, with insomnia and reliving the event, a situation also found in our study but as an isolated incident.

As Verhoef et al. showed as well [5], the period of investigation can leave a significant mark in the personal lives of many of the doctors involved, even if some can cope more easily, without feeling the event as a major emotional burden. For some of the participants in our study, the professional impact of the complaint was not perceived as major, either because they were convinced that they did everything they could to save the patient or because previous complaints had led them to look with detachment on such conflicts. This last situation is also captured by Jain and Ogden [16], who reported that some of the doctors may become “immune” to complaints.

The feeling of insecurity towards medical practice, with the decrease of confidence in their own medical knowledge as a result of the complaint, was common in our study and other studies in the literature [16,17,21,22], often persistent over time even in cases that are not very difficult to manage [31]. This feeling is manifested by a particular approach to their own practice by the accused doctors, materialized by requesting additional consultations, excessive caution, excessive checks and frequent questions about the correctness of the medical activities performed [13,16].

Both the feeling of insecurity and the strong negative emotional impact escalate over time into permanent changes in practice. The fear of a new complaint is a common theme found in the literature [5]—doctors performing their activities under the threat of this possibility [31].

In the process of making diagnostic and therapeutic decisions, the doctor must filter his/her medical knowledge through the prism of professional responsibility, which accompanies any medical act [27]. Through professional commitment, the doctor is available to the patient with all his/her knowledge and abilities [22]. When the latter turns against the doctor—complaining if the result of the medical act does not coincide with their expectations—there is a risk that the doctor will develop a defense mechanism that no longer meets the standards of professionalism, triggering an imbalance between medical thinking and legal issues of the medical practice [22,32]. This creates the premises for defensive medical practice.

In simple terms, defensive medicine refers to the practice of the medical profession aiming to protect the doctor from complaints from patients to the detriment of rational clinical and scientific thinking, which has as its primary purpose the best interest of the patient [16,27,33,34]. Thus, the defensive practices imply both excessive investigations, consultations or medication [2,30,35] and avoiding patients with conditions that require complex interventions, avoiding or early abandonment of complex procedures or even changing the specialty or giving up the medical profession [2,30,34].

Complaints, defensive practices and poor doctor–patient relationships are interconnected. On the one hand, the complaint may be the result of an altered doctor–patient relationship, and on the other hand, one of the consequences of complaints is the alteration of the doctor–patient relationship [30] when doctors resort to defensive practices and decide to distance themselves from patients, as our study showed.

Some doctors see in supplementing the investigations a way to prove—in the event of a complaint—that they have fulfilled their duty with diligence and prudence [8,36] and not a lack of professionalism [22]. Others appreciate that resorting to such practices is not an option that the physician chooses from many available, but the only option he/she has [22] in a world dominated by complaints.

Defensive medicine practices are growing in direct correlation to complaints and are being adopted by all medical systems [27], excessive investigations and medicines being a common problem in many Western countries [27,37]. An example in this regard is Italy, the European country with the highest rate of malpractice complaints against doctors in 2009, where doctors perform their practice by overusing hospital resources as a means of defending against complaints [36].

The most prone to such practices are doctors with a history of complaints [2,33], who have suffered significant emotional disorders [2] and who are afraid to go through all the suffering caused by the investigation process in the event of a new complaint [5]. However, looking at the phenomenon of defensive practices as a whole, it can be noticed that the implications of the complaints extend in multiple directions and target the accused doctor and also the patients, the medical system [10,38,39], the colleagues of the doctors involved in complaints [5,27] and even the training of future doctors [30].

In our study we identified both supplementary defensive practices, in the sense of requesting more investigations and referring to more consultations, and avoidance defensive practices, such as giving up complex interventions or avoiding interventions in complicated cases. Practices of limiting medical services in response to complaints were also identified by Jain and Ogden [16], one of the doctors interviewed in that study considering that sometimes he was too involved in helping patients. We identified similar results in studies conducted in Italy. For example, in the study conducted by the Federico Stella Center, 51.8% of respondents said that experiencing complaints of malpractice changed their perception of how to approach patients and made them determined to apply defensive practices [27]. In the study conducted by Catino and Locatelli, cited by Toraldo et al. [27], 34.3% of participants reported the same changes. Nahed et al. [40], who conducted a study involving 3344 neurosurgeons, reported that 45% of the participants gave up risky procedures, and Studdert et al. [41] identified the same avoidance trends in 42% of the 824 physicians participating in their study.

Most often, such practices are detrimental to the quality of patient care and patient safety and to the medical system through the significant additional costs generated by unnecessary investigations [30,34,35], which could be used for situations where they are really needed. In addition, the quality of care and patient safety could suffer from the shortage of specialists in case of resignation [25] or early retirement [13,34,42], especially in specialties with a general shortage of doctors [35]. Thus, the purpose of defensive practices is the protection of physicians, while patients can become indirect victims [2,10], either by conducting unnecessary diagnostic investigations [22,34] or by avoiding complex and risky life-saving procedures [34,43]. In this regard, there are studies to show that supplementary practices (such as over-prescribing investigations and medication) are not clinically effective and do not bring additional benefit to the patient, the advantage remaining only the legal protection of the doctor against complaints from patients [27,44]. At the opposite pole are avoidance practices, and Debono et al. [31] showed that 60.2% of the 78 French neurosurgeons who work in the private sector and who responded to an online questionnaire refused to treat patients who needed risky interventions because they feared a complication and a subsequent complaint, although experience and competence would have enabled them to carry out those interventions. Bourne et al. [3] showed that 49.8% of the 2257 physicians involved in an investigation procedure for ongoing or recent complaints and 42.95% of the 3889 physicians who have faced complaints in the more distant past avoid risky procedures or patients with complex diseases, and 14% of all respondents consider the possibility of early abandonment of complex procedures.

The thoughts of changing the profession among the doctors participating in our study were transient, without special significance, unlike the studies published by Bourne et al. [2], Jain and Ogden [16] and Wallace et al. [34], the latter stating that the psychological stress associated with complaints caused more than a third of the doctors involved to consider early retirement or a change of profession [34].

Still, as a defensive way of practicing medicine, we found among the doctors in our study the supplementation of the information provided to patients and the caution with which they record the information in medical files. This way of protection is also found in other studies. For example, 74% of physicians who participated in the study conducted by Reed et al. [30] reported that the fear of complaints causes doctors to spend more time filling medical files, as they are aware that in order to defend against a possible complaint the information in the medical file is objective evidence for the treatment they indicated and for monitoring the evolution of the health status of the patient or even for the information that the patient disclosed to the doctor [36,45].

As mentioned by one of the doctors participating in our study, as well as doctors involved in other studies, the fear of complaints can induce these practices among colleagues who were not accused but who witnessed the implications of complaints on their colleagues [5]. Thus, in the study conducted by the Provincial Order of Physicians of Rome, cited by Toraldo et al. [27], 48.4% of respondents stated that they have transformed regular medical practice into a defensive practice as a result of the impact of malpractice complaints faced by their colleagues. In the study conducted by the Federico Stella Center, cited by Toraldo et al. [27], the same result was identified in 65.7% of participants.

An important change in the practice of the doctors participating in our study was at the relational level, in the sense of improving communication with the patients and their families. One of the doctors shared regret about the role he believes the lack of communication had in triggering the complaint. Some of the opinions of the doctors involved in our study are consistent with previous studies or general data published in the literature, which universally recognized the importance of an effective relationship between doctor and patient/family in the success of the medical act or, conversely, poor relationship triggering complaints [1,26,36], communication being recognized as the basis of an effective relationship. Schaad et al. [10] conducted a study analyzing the reasons why patients and their relatives visit the hospital’s complaints center and found that the reasons related to the relational aspects of the doctor–patient binomial (e.g., unanswered questions, lack of mutual understanding, unbalanced relationship, breach of confidentiality) were significantly more reported compared to the technical aspects of medical practice (e.g., lack of diagnosis, failed surgeries). Similarly, there are other studies showing that in the process of initiating the complaint, prevailing aspects included poor and/or incomplete communication, lack of honesty and respect [46,47,48], leaving in the background the medical act itself (e.g., care, treatment, documentation) [1,48].

Just as there is evidence that poor communication increases the risk of complaints, there is also evidence that improved communication can prevent complaints despite the patient or his/her family finding a medical error [49]. By improving communication, the doctor has the opportunity to inform the patient more clearly about the risks and benefits of a particular method of diagnosis or treatment, so that the patient accepts or rejects them fully aware of the clinical situation [35]. This information process is materialized in the conscious signing of the informed consent form by the patient or by his/her legal representative.

Changes in doctor–patient communication as a result of complaints can be found in other studies published in the literature, with doctors becoming more aware of the importance of effective communication with the patient by repeating information, checking their understanding of information, and recognizing that lack of communication is in fact, poor communication [10].

Another aspect highlighted by the doctors in our study is the link between complaints and the low level of medical education of the population. Thus, many of the complaints arise because patients do not understand medical terms, priorities or medical procedures. The lack of education is maintained by erroneous information in the virtual environment or in the media and indirectly contributes to non-adherence to medical recommendations or delayed presentation to the doctor, thus creating the premises for therapeutic failure and for the subsequent complaint. A similar reason for complaints was reported by Schaad et al. [10]; the doctors participating in their study stated that sometimes patients’ complaints were related to the limits of medicine or stemmed from the patients’ disappointment with medicine in general, especially from the feeling that the medical team did not do everything possible to resolve the case. In response to this type of complaint, some physicians in the study by Schaad et al. [10] made changes in their practice to provide patients with detailed information about medical procedures and about their health, in order to prevent unrealistic expectations, an idea found in our study as well.

Regarding the postponement of the presentation to the doctor, the literature proposes another hypothesis for the reasons that determine the initiation of the complaint: the search for a scapegoat. The situations presented by the doctors in our study are cases that ended with the death of the patients and the families blamed the doctors, saying that they made mistakes or did not do everything possible to save them, though the patients ignored the recommendations of the doctors and did not seek medical help in due time. The scapegoat hypothesis is supported by Davis and Scott [50], who state that patients’ relatives file complaints to get rid of guilt. For example, because even though they noticed that the patient’s health was deteriorating, they did not ask for medical help in due time. Therefore, in order to free themselves from the burden of guilt, they seek to blame the doctors, claiming that they did not do everything possible to save the patient’s life [50].

## 5. Conclusions

Our study revealed that physicians who have received formal complaints from patients are deeply affected by these accusations and the investigation procedure. For many doctors, the impact during the investigation was dominated at a personal level by insomnia, nightmares and stress and anxiety, and at a professional level by doubts about medical decisions, fear, anxiety and the tendency to avoid patients, especially those with severe pathologies.

To a lesser extent, for other physicians, the experience of the complaint was less significant—either because of the belief that they did everything in their power to save the patient or because the experience of previous complaints has made them approach the complaints with detachment.

The professional impact following the complaint materialized in changes regarding the doctor–patient relationship, emphasizing the process of providing information to the patients, but doctors also reported the adoption of defensive medicine practices, including more careful recording of medical data.

Our study also showed the need for changes in the legal system in the sense of clearer legislation on medical malpractice and also for changes in the medical system in order to create mechanisms to support accused doctors during the course of the investigation process.

### Future Practical Implications

The results of this qualitative research can be the starting point and the basis of recommendations or guidelines with wide applicability, to improve the quality and safety of patient care and to reduce the personal and professional impact of complaints on doctors. Moreover, the results of our study can be a motivation for changes in the legal and medical systems, designed to protect the accused doctors and ensure a high quality for medical practice, for example, by refining the extrajudicial procedure for the analysis of the patients’ complaints, organizing training sessions on medical topics for the legal professionals involved in the analysis of complaints and basic medical education for the general population. Hospital management could provide legal assistance for the doctors involved in complaints, to make it easier for them to cope with the stress and anxiety related to the legal aspects of the complaint. All these measures could reduce the impact of the complaints on the doctors, leading to a safer practice both for the patients and the doctors, increasing at the same time the quality of the medical act.

Moreover, *future research* could target the analysis of the views of both the patient and the doctor involved in a specific case. In-depth analysis of the consequences of the complaints on more doctors in the same specialty and in the same geographical area could reveal other important topics to work on in order to reduce the number of complaints.

*Strengths and limitations*. This qualitative research allowed in-depth exploration of the personal and professional experiences of physicians who experienced a complaint from patients. However, given the qualitative design, the results obtained cannot be generalized. Another limitation of our study is the fact that we could not cover more specialties, to have an in-depth analysis in more areas, even though the participants came from all three major categories of specialties (surgical, medical and paraclinical). Nevertheless, the results of this study provided the authors with in-depth knowledge of the impact of professional liability complaints on doctors and contributed to a large extent to the creation of a questionnaire that was widely applied to doctors in Romania and allowed the achievement of generalizable quantitative results on this topic.

## Data Availability

The data presented in this study are available on request from the corresponding author. The data are not publicly available due to privacy and ethical issues.
